# Chronic Treatment with *Nigella sativa* Oil Exerts Antimanic Properties and Reduces Brain Inflammation in Rats

**DOI:** 10.3390/ijms25031823

**Published:** 2024-02-02

**Authors:** Sarit Uzzan, Ira-Sivan Rostevanov, Elina Rubin, Olivia Benguigui, Said Marazka, Jacob Kaplanski, Riad Agbaria, Abed N. Azab

**Affiliations:** 1 Department of Clinical Biochemistry and Pharmacology, Faculty of Health Sciences, Ben-Gurion University of the Negev, Beer-Sheva 8410501, Israelriad@bgu.ac.il (R.A.); 2 Department of Kinesiology and Physical Education, McGill University, 475 Pine Avenue West, Montreal, QC H2W1S4, Canada; 3 Department of Cognitive and Brain Sciences, Faculty of Humanities and Social Sciences, Ben-Gurion University of the Negev, Beer-Sheva 8410501, Israel; 4 Department of Nursing, Faculty of Health Sciences, Ben-Gurion University of the Negev, Beer-Sheva 8410501, Israel

**Keywords:** *Nigella sativa*, mania, inflammation, behavior, mental illness

## Abstract

*Nigella sativa* (NS) is a native herb consumed habitually in several countries worldwide, possessing manifold therapeutic properties. Among them, anti-inflammatory features have been reported, presumably relating to mechanisms involved in the nuclear factor kappa-B pathway, among others. Given the observed association between neuroimmune factors and mental illness, the primary aim of the present study was to examine the effects of chronic NS use on manic-like behavior in rats, as well as analyze levels of brain inflammatory mediators following NS intake. Using male and female rats, baseline tests were performed; thereafter, rats were fed either regular food (control) or NS-containing food (treatment) for four weeks. Following intervention, behavioral tests were induced (an open field test, sucrose consumption test, three-chamber sociality test, and amphetamine-induced hyperactivity test). Subsequently, brain samples were extracted, and inflammatory mediators were evaluated, including interleukin-6, leukotriene B4, prostaglandin E2, tumor necrosis factor-α, and nuclear phosphorylated-p65. Our findings show NS to result in a marked antimanic-like effect, in tandem with a positive modulation of select inflammatory mediators among male and female rats. The findings reinforce the proposed therapeutic advantages relating to NS ingestion.

## 1. Introduction

*Nigella sativa* (NS) is a native herb of Southwest Asia, North Africa, and Southern Europe, also cultivated in Mediterranean and Middle Eastern countries [1]. NS is used worldwide both as a garnish in cuisine and as a therapeutic in traditional medicine. Its seeds and oil carry an age-old history of remedial use, particularly in Islamic culture [2,3]. NS displays various pharmacological properties [4], demonstrating therapeutic effects among subjects with dyslipidemia, diabetes mellitus and cardiovascular disorders [3,5,6,7]. Moreover, studies have found NS to possess anticonvulsant [8], antidepressant [9,10], anxiolytic [11], anti-ischemic [12], and analgesic effects [13,14]. Additionally, NS may protect against neurodegenerative conditions such as Alzheimer’s [15] and Parkinson’s disease [16]. Pertinent to the present study, NS has repetitively exhibited anti-oxidative and anti-inflammatory properties [17,18,19,20,21], reducing the symptoms of headaches, menstrual cramps, and arthritis [22] and boosting immunity [23,24]. Several studies reported NS, and its active component thymoquinone, to reduce levels of pro-inflammatory cytokines including interleukin (IL)-6 and tumor necrosis factor (TNF)-α [3,25,26,27,28,29].

Evidence shows that NS suppresses nuclear factor kappa-B (NF-κB) activity [30,31]; several components of innate and adaptive immune processes are mediated by the NF-κB transcription factor. NF-κB modulates the activation, differentiation, and survival of innate immune and inflammatory T-cells and incites the expression of several pro-inflammatory cytokines and chemokines. Alterations in NF-κB activity have been observed to be pathogenically relevant in certain inflammatory conditions [32,33,34]

**Figure 1:**
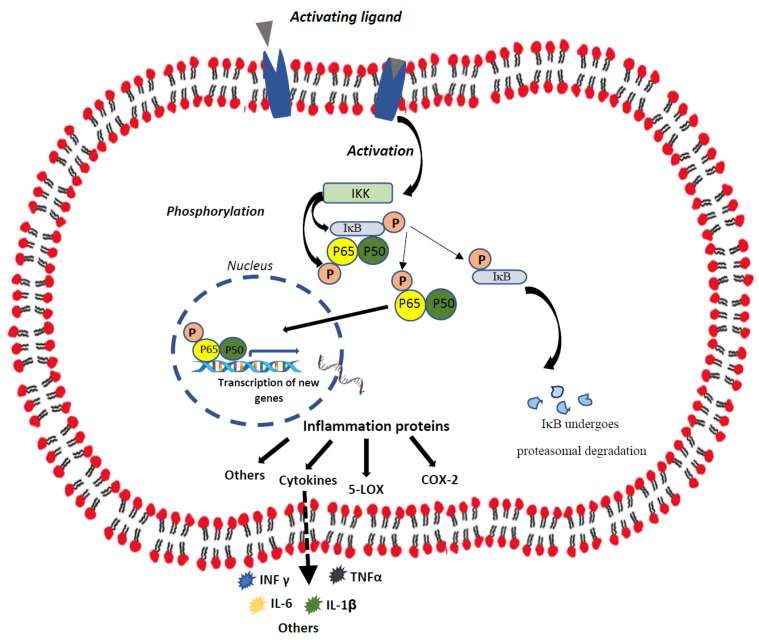
Illustration of the NF-κB pathway. The NF-κB heterodimer consisting of p65 and p50 resides in the cytosol, complexed with the inhibitory protein IκB. A variety of extracellular signals activate the enzyme IKK, which, in turn, phosphorylates IκB, resulting in the dissociation of IκB from NF-κB proteins. IKK also phosphorylates the p65-p50 heterodimer, leading to its migration into the nucleus where it binds to specific sequences of the DNA. The NF-κB-DNA complex regulates different gene transcriptions and promotes inflammation and other cellular processes. Abbreviations: COX, cyclooxygenase; IκB, inhibitor κB; IKK, IκB kinase; IL, interleukin; INF, interferon; LOX, lipoxygenase; TNF, tumor necrosis factor.

NF-κB, a protein complex governing cytokine production and cell survival [32,35,36], is found in almost all animal cell types and is involved in cellular responses to stimuli such as inflammatory cytokines, metabolic stress, and ischemia. NF-κB belongs to the Rel family of transcription factors and includes p50, p65, c-rel, and RelB, which are relevant in healthy as well as pathologic processes [36]. The activated NF-κB subunits assemble to form homo- or heterodimers transcription factor complexes displaying a DNA-binding ability and transactivation potential. The NF-κB dimers remain inactive in the cytoplasm, with IκΒ inhibitory proteins masking the nuclear localization sequence and, thus, blocking their translocation to the nucleus [37]. The phosphorylation of IκB by IκB kinase (IKK) releases the p65:p50 dimer from the inhibitory complex and facilitates the degradation of IκB in the proteasomes (see Figure 1 for illustration). The induced activation of NF-κB in response to a variety of stimuli is typically transient, but sufficient to upregulate the transactivation of target genes of diverse activities [38]. The activation of NF-κB is associated with the prominent release of pro-inflammatory cytokines such as IL-1β, IL-6, interferon-γ, and TNF-α [39,40].

NF-κB regulates gene expression in the arachidonic acid-eicosanoid pathway, including the inflammation-associated enzymes 5-lipoxygenase (LOX) [41] and cyclooxygenase (COX)-2 [42,43]. 5-LOX catalyzes the formation of leukotrienes (LTs)—inflammatory constituents involved in various immune–inflammatory–allergic responses [44,45]. One of the most active LTs in mammalian tissues is LTB4; it facilitates the immune response by activating leukocytes and acting as a potent pro-inflammatory mediator in peripheral tissues and the brain [45,46]. COX-2 catalyzes arachidonic acid to produce prostaglandins (PGs) [45]. PGE2 is one of the most abundant PGs in mammals, exhibiting versatile biological functions both during physiological and pathological conditions [47]. Numerous studies have demonstrated that PGE2 is produced and secreted by glia cells [48,49,50], and a large body of data attests to a link between alterations in PGE2 levels and mood disorders [51,52].

Thymoquinone, a major active compound found in NS seeds, has been studied for its potential health benefits and has conferred anti-inflammatory, antioxidant, and immune-boosting effects [53,54,55,56]. Pertinent to the present study, former studies suggested NS may influence hypothalamus (HT) activity. The HT is responsible for regulating many bodily functions, including appetite, thirst, sleep, and the release of hormones that control growth and development [57]. In animal studies, NS displayed influence on the production of hormones that regulate hunger and metabolism, suggesting possible impact on the HT [53]. Bargi et al. [58] found that thymoquinone improved learning and memory impairments in rats by attenuating brain cytokine levels and oxidative damage. Additionally, thymoquinone has neuroprotective effects against epilepsy, parkinsonism, and anxiety, and improved learning and memory [59]. Furthermore, thymoquinone has exhibited beneficial behavioral effects in rodents [11,60,61,62]. For example, Kadil et al. [60,61] demonstrated an NS treatment that resulted in a significant antidepressant-like effect in rats exposed to an unpredictable chronic mild stress procedure.

In recent decades, there has been appreciable focus on the immune system, inflammatory components, and their interconnection with mental disorders, depression in particular, finding that the disruption of certain cytokine signaling is present in these conditions [63,64]. Elements involved include TNF-α, IL-6 and, NF-κB, among several others [65,66,67,68,69,70,71,72,73,74,75,76,77]. Moreover, psychotropic drugs evidently possess anti-inflammatory attributes and, in parallel, anti-inflammatory medications have shown beneficial outcomes in psychiatric illness treatments [74,78,79,80,81,82,83,84,85,86].

As stated, NF-κB has relevantly shown prominent involvement in pathobiological mechanisms and the treatment of psychiatric illness [87,88,89,90,91,92]. Nevertheless, a methodic investigation on NF-κB activity and NS in mania-like models has not been adequately explored. Therefore, the primary aim of this study was to examine the effects of chronic treatment with NS oil on behavioral phenotypes, particularly manic-like phenotypes, in rats. Additionally, we examined the effects of NS treatment on brain inflammatory mediator levels.

## 2. Results

### 2.1. Chronic NS Treatment Exerts Positive Behavioral Outcomes in Rat Models of Depression and Mania

#### 2.1.1. Sucrose Consumption

**Figure 2:**
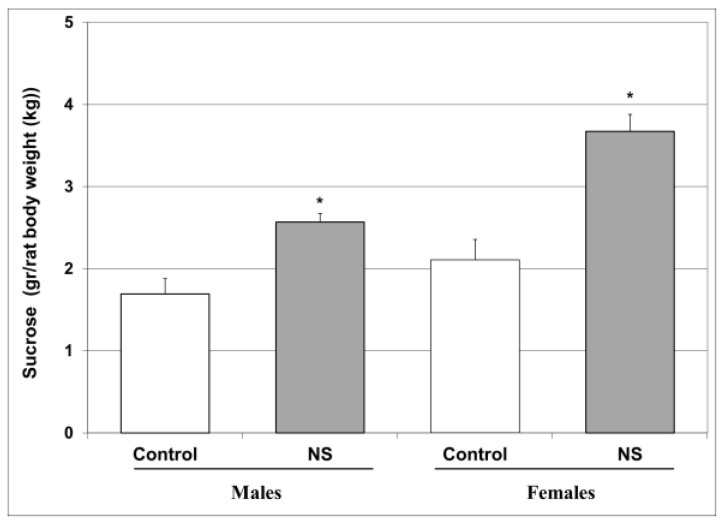
Effect of NS on sucrose consumption. Rats were fed regular food (control) or NS-containing food (NS) for four weeks. Then, rats were subjected to a sucrose consumption test for 24 h. Results represent the mean ± SEM of 24 rats per group. One-way ANOVA: F = 19.49, *p* < 0.0001; Post Hoc Least Significant Difference (LSD): Males—* *p* = 0.001 vs. control; females—* *p* = 0.0002 vs. control.

At baseline, there were no significant differences in sucrose consumption between all study groups. Chronic treatment with NS for four weeks significantly increased sucrose consumption in male and female naïve rats as compared to vehicle-treated animals (Figure 2), suggesting a hedonic-like effect of NS.

#### 2.1.2. Social Interaction

**Figure 3:**
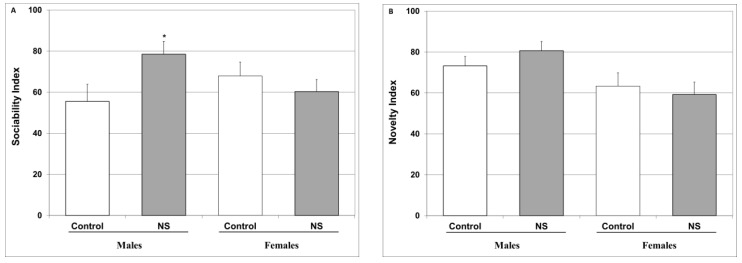
Effect of NS on social interactions. Rats were fed regular food (control) or NS-containing food (NS) for four weeks. Then, rats were subjected to a TCT to assess sociability (**A**) and social novelty (**B**). Results represent the mean ± SEM of 23–24 rats per group. (**A**) One-way ANOVA: F = 3.085, *p* = 0.03; Post Hoc LSD: Males—* *p* = 0.036 vs. control; females—*p* > 0.05 vs. control. (**B**) One-way ANOVA: F = 2.038, *p* = 0.11; Post Hoc LSD: *p* > 0.05 in males and females.

Figure 3A shows that chronic treatment with NS significantly increased the sociability index in male rats. On the other hand, NS treatment did not alter the sociability index in female rats (Figure 3A) and did not affect the social novelty index in either sex (Figure 3B).

#### 2.1.3. Hyperactive/Mania-Like Behavior

**Figure 4:**
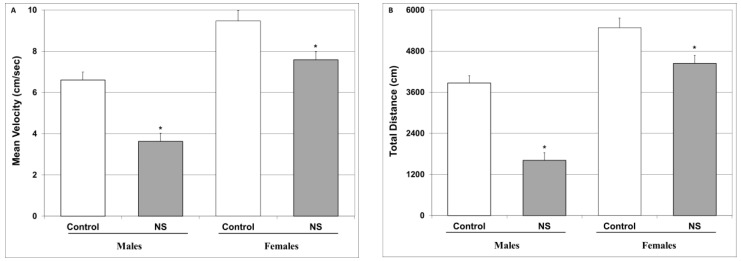
Effect of NS on amphetamine-induced hyperactivity. Rats were fed regular food (control) or NS-containing food (NS) for four weeks. On the AIHT day, rats were injected with amphetamine, after which, they were placed in an open field arena for 30 min. The mean velocity (**A**) and total distance traveled (**B**) during the last 20 min of the sessions were assessed using a video-tracking system. Results represent the mean ± SEM of 24 rats per group. (**A**) One-way ANOVA: F = 33.45, *p* < 0.0001; Post Hoc LSD: Males—* *p* < 0.00001 vs. control; females—* *p* = 0.0051 vs. control. (**B**) One-way ANOVA: F = 46.76, *p* < 0.0001; Post Hoc LSD: Males—* *p* < 0.00001 vs. control; females—* *p* = 0.0064 vs. control.

Similar to previous studies [93,94], one day before the AIHT (see Section 4.3.4), rats were subjected to an OFT session (Section 4.3.2) to measure their spontaneous locomotor activity in order to detect any variations or abnormalities. The analysis revealed that there were no significant differences between the experimental groups regarding the velocity of movement and total distance traveled (*p* values were greater than 0.05 for all comparisons between the control and NS-treated male and female rats): velocity—males: 3.64 ± 0.28 vs. 3.01 ± 0.23, females: 3.92 ± 0.22 vs. 4.3 ± 0.23; distance—males: 3205 ± 266 vs. 2932 ± 246, females: 4085 ± 297 vs. 4435 ± 271. On the next day, amphetamine was administered to all groups to trigger hyperactive/mania-like behavior. The administration of amphetamine significantly elevated the mean velocity and distance traveled in the male and female control animals as compared to the previous day (values are mean ± SEM): velocity—males: 6.60 ± 0.39 vs. 3.58 ± 0.28, *p* < 0.001, females: 9.47 ± 0.50 vs. 3.75 ± 0.27, *p* < 0.001; distance—males: 3867 ± 216 vs. 3244 ± 264, *p* = 0.08, females: 5485 ± 280 vs. 4002 ± 322, *p* = 0.001). As seen in Figure 4, chronic treatment with NS significantly decreased the mean velocity (Figure 4A) and distance traveled (Figure 4B) both in male and female rats, indicating an anti-hyperactive/anti-manic-like effect of NS.

### 2.2. Chronic NS Treatment Modulates the Levels of Inflammatory Mediators in Rat Brain

#### 2.2.1. TNF-α and IL-6

**Figure 5:**
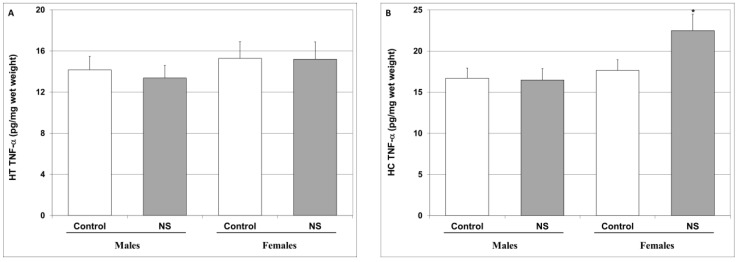

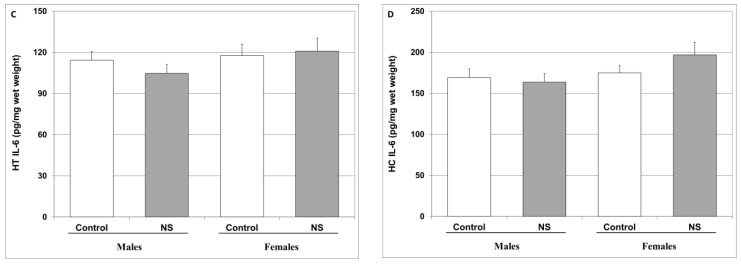
Effect of NS on brain TNF-α and IL-6 levels. Rats were fed regular food (control) or NS-containing food (NS) for four weeks. On the last day of the experimental protocol, rats were euthanized, and their brains ousted and processed as described in Section 4. TNF-α (**A**,**B**) and IL-6 (**C**,**D**) levels in the HT (**A**,**C**) and HC (**B**,**D**) were determined via ELISA. Results represent the mean ± SEM of 20 samples per group. (**A**) One-way ANOVA: F = 0.368, *p* = 0.77; Post Hoc LSD: *p* > 0.05 in males and females. (**B**) One-way ANOVA: F = 3.51, *p* = 0.019; Post Hoc LSD: males—*p* > 0.05; females—* *p* = 0.048 vs. control. (**C**) One-way ANOVA: F = 0.765, *p* = 0.516; Post Hoc LSD: *p* > 0.05 in males and females. (**D**) One-way ANOVA: F = 1.58, *p* = 0.2; Post Hoc LSD: *p* > 0.05 in males and females.

As seen in Figure 5, in the present study, we found that NS did not significantly alter TNF-α or IL-6 in the HT or HC of both sexes, except for an increase in the HC TNF-α levels in female rats (Figure 5B).

#### 2.2.2. PGE2 and LTB4

**Figure 6:**
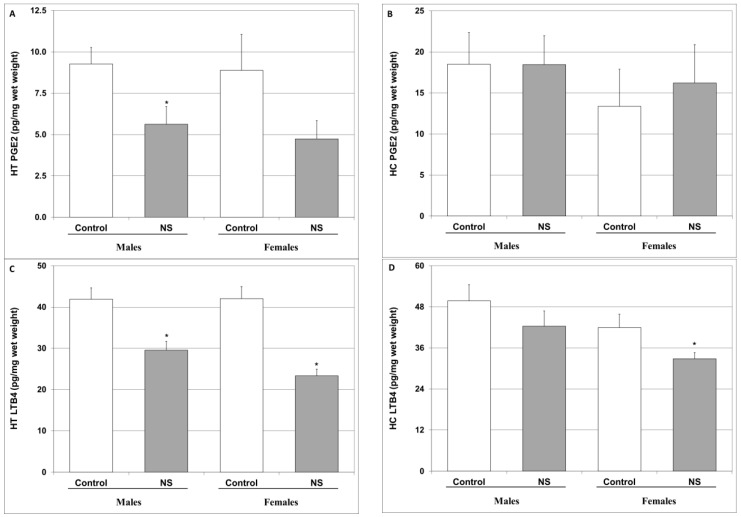
Effect of NS on brain PGE2 and LTB4 levels. Rats were fed regular food (control) or NS-containing food (NS) for four weeks. On the last day of the experimental protocol, rats were euthanized, and their brains ousted and processed as described in Section 4. PGE2 (**A**,**B**) and LTB4 (**C**,**D**) levels in the HT (**A**,**C**) and HC (**B**,**D**) were determined via ELISA. Results represent the mean ± SEM of 10 (PGE2) or 20 (LTB4) samples per group. (**A**) One-way ANOVA: F = 2.78, *p* = 0.05; Post Hoc LSD: Males—* *p* = 0.022 vs. control. (**B**) One-way ANOVA: F = 0.34, *p* = 0.74; Post Hoc LSD: *p* > 0.05 in males and females. (**C**) One-way ANOVA: F = 15.02, *p* < 0.0001; Post Hoc LSD: males—* *p* = 0.001 vs. control; females—*p* < 0.00001 vs. control. (**D**) One-way ANOVA: F = 3.008, *p* = 0.03; Post Hoc LSD: males—*p* > 0.05 vs. control; females—* *p* = 0.048 vs. control.

As depicted in Figure 6, chronic NS treatment significantly decreased HT PGE2 levels in male rats and led to a nearly significant reduction in females (Figure 6A). In contrast, NS did not alter PGE2 levels in the HC (Figure 6B). Furthermore, NS significantly attenuated the LTB4 levels in the HT (Figure 6C), whereas in the HC, it had a prominent effect only in female rats (Figure 6D).

#### 2.2.3. Nuclear P-p65

**Figure 7:**
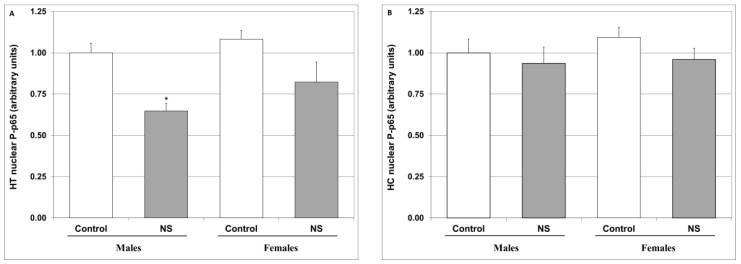
Effect of NS on brain nuclear P-p65 levels. Rats were fed regular food (control) or NS-containing food (NS) for four weeks. On the last day of the experimental protocol, rats were euthanized, and their brains ousted and processed as described in Section 4. Nuclear P-p65 levels in the HT (**A**) and HC (**B**) were determined via ELISA. Results represent the mean ± SEM of 20 samples per group. (**A**) One-way ANOVA: F = 5.504, *p* = 0.001; Post Hoc LSD: males—* *p* = 0.00002 vs. control; females—*p* > 0.05 vs. control. (**B**) One-way ANOVA: F = 0.78, *p* = 0.5; Post Hoc LSD: *p* > 0.05 in males and females. P-p65 denotes phosphorylated p-65.

In order to try and elucidate the mechanism underlying the observed anti-inflammatory effects of NS, as seen in Figure 6 (reductions in the PGE2 and LTB4 levels in rat brain), we examined the effect of NS on the nuclear levels of P-p65. As seen in Figure 7A, NS significantly decreased the nuclear P-p65 levels in the HT of male rats and led to a non-significant decrease in female rats too. Conversely, NS treatment did not influence P-p65 levels in the HC (Figure 7B).

## 3. Discussion

The main objective of the present study was to examine the anti-manic-like potential of NS. We found that NS exerts potent anti-manic/hyperactive-like effects in male and female rats. Moreover, similar to previous findings, we found that chronic NS treatment is associated with a hedonic-like effect. Importantly, the observed positive behavioral effects of NS were accompanied by a significant reduction in the brain levels of several inflammatory mediators.

Previous studies that investigated the behavioral effects of NS in animal models mainly focused on its antidepressant and anxiolytic potential revealing positive therapeutic outcomes [9,11,61,62,95]. In the present study, we found that chronic treatment with NS significantly increased sucrose consumption in male and female naïve rats (Figure 2), which is suggestive of a potent hedonic-like effect. Together with the results of previous studies in animals [9,11,61,62,95], our findings further strengthen the pro-hedonic/antidepressant potential of NS treatment. A strong testimony to the antidepressant potential of NS came from a randomized clinical trial of patients with depression [96]. In a randomized, double-blind, placebo-controlled trial, Zadeh et al. [96] showed that NS treatment significantly decreased the severity of depressive symptoms, while significantly increasing the serum levels of the neuroprotective protein brain-derived neurotrophic factor. The precise mechanism underlying the antidepressant effect of NS is currently unknown. Perveen et al. [95] demonstrated that chronic NS treatment resulted in a significant antidepressant-like effect, which was accompanied by an increase in serotonin levels in the rat brain. Consistently, NS has been shown to increase serotonin levels in other animal studies [62], which is supportive of a possible mechanistic explanation for its antidepressant-like and anxiolytic-like properties.

Accumulating data suggest that NS treatment improves cognitive function in animals and human subjects [62,97,98]. On the other hand, we are not aware of previous studies that focused on the social behavioral aspects of NS treatment. Therefore, in the present study we examined the effects of chronic NS treatment on the sociability and social novelty of the experimental rats utilizing the three-chamber test (TCT). The results show that NS does not exhibit a prominent influence on the social behaviors of naïve rats (Figure 3). Clearly, more studies are needed to elucidate the social behavioral outcomes of chronic NS treatment.

As mentioned above, the primary aim of the present study was to investigate the anti-manic-like potential of NS. Utilizing a widely accepted animal model for inducing mania-like behavior—the amphetamine-induced hyperactivity test (AIHT)—we demonstrated that chronic NS treatment significantly reduced the mean velocity and total distance traveled in amphetamine-injected male and female rats (Figure 4), which is indicative of a potent anti-manic/anti-hyperactive-like effect. To the best of our knowledge, this is the first study to show that chronic NS treatment—under experimental conditions mimicking the “regular” chronic consumption of NS in humans—significantly attenuates mania-like behavior in rodents. A study by Roohbakhsh et al. [99] tested the effects of NS on methamphetamine-induced hyperlocomotion in mice. It showed that NS (thymoquinone) diminished the increase in total distance traveled in methamphetamine-treated animals. Nevertheless, the aims and experimental design of that study were different from those of our study. For example, in comparison to the present study, that of Roohbakhsh et al. [99] used purified thymoquinone (rather than using NS oil/seeds), which was injected (not provided in the food) at relatively high doses (5–20 mg/kg). Moreover, they used an experimental protocol in which methamphetamine was administered repetitively (four times a day, 2 h apart; it is not clear for how many days). The repetitive administration of amphetamine may induce tolerance to the stimulating effect of the drug and thus result in entirely different behavioral phenotypes [100,101]. Additionally, the highest used dose of thymoquinone—20 mg/kg—caused an increase in locomotion in the central zone of the open field arena, a result which may actually attest to a pro-manic-like behavior. Importantly, the administration of 20 mg/kg of thymoquinone to naïve (non-methamphetamine-treated) mice led to a significant decrease in total distance traveled, which is suggestive of a possible toxic effect of this dose on animals’ behavior. Our finding that NS exhibits an anti-manic/anti-hyperactive-like effect further establishes the beneficial behavioral profile of NS and, at the same time, underscores the need to replicate these findings in future animal as well as human studies. Of note, in the present study we did not determine which chemical component(s) of the NS oil in particular (e.g., thymoquinone) is/are responsible for its anti-manic-like effect or other behavioral outcomes. Although thymoquinone is recognized as the most active chemical component of NS, responsible for most of its pharmacological and toxicological effects [7,53,54,55,56,102], it is possible that other chemical components of NS, such as polyunsaturated fatty acids, contribute to the beneficial outcomes. However, the present study did not aim to identify which specific component of NS induced the observed pharmacological/behavioral outcomes, but rather to examine NS oil “as is”, in the form consumed by lay people within routine nutritional consumption and explore its potential medicinal/therapeutic properties. The therapeutic mechanism of the anti-manic-like effect of NS is still obscure. The results of a previous in vitro study [103] may hint at a possible mechanism. It demonstrated that the treatment of cultured neurons with an NS extract significantly increased the level of the inhibitory neurotransmitter gamma-amino-butyric acid (GABA), while decreasing the levels of the excitatory neurotransmitters glutamate and aspartate [103]. If NS treatment leads to similar outcomes in vivo in the brain of treated subjects, this may explain its anti-hyperactive/anti-manic-like effect.

Many studies that have been published during the last decade reinforce the relationship between inflammation and mood disorders [104,105,106,107,108,109]. As mentioned earlier, numerous studies demonstrated alterations in IL-6 and TNF-α levels in patients with depression [110,111], bipolar disorder, schizophrenia [110,112,113], and other psychiatric illnesses [105]. Furthermore, many studies have suggested that psychotropic drugs exhibit strong anti-inflammatory effects that may impact and possibly enhance their therapeutic efficacy [114,115,116,117].

In the present study, we initially examined the effects of NS on IL-6 and TNF-α levels, owing to the strong association between these cytokines and mental illness. Surprisingly, and in contrast to previous studies that reported a reduction in IL-6 and TNF-α levels under treatment with NS [118,119], we found that, overall, chronic NS treatment did not significantly alter IL-6 and TNF-α levels in the HT and HC of treated rats (Figure 5). Two reasons may account for these findings: first, the present study used a relatively low dose of NS—250 mg/kg/day, in comparison to previous studies that used a chronic NS treatment regimen in vivo dose-range between 100 and 2500 mg/kg/day [9,27,29,97,120]. The choice to use such a low dose of NS derived from our will to mimic an “authentic” practically-occurring consumption regimen in humans. Second, former studies that tested the effects of chronic NS treatment on IL-6 and TNF-α levels under non-stimulated/normal conditions did not determine their levels in particular brain regions (e.g., HT and HC), similar to the present study.

Another inflammatory pathway that is frequently linked to the pathophysiology and treatment of psychiatric disorders is that of the arachidonic acid-eicosanoid cascade [45,121,122,123]. Importantly, numerous randomized clinical trials have shown that COX-inhibitor add-on therapy in psychiatric medications is beneficial for the treatment of various mental illnesses [121,123]. Interestingly, we found that chronic NS treatment significantly attenuated HT PGE2 levels in male rats and led to a nearly significant decrease in female rats (Figure 6A). This is in line with previous findings attesting to the ability of NS to inhibit COX and reduce PGE2 production [124,125]. Moreover, similar to the results of previous studies [126,127], we found that, NS treatment diminished LTB4 levels in the HT and HC of male and female rats (Figure 6C,D). Taken together, these findings suggest that NS exerts a potent anti-inflammatory influence in the arachidonic acid-eicosanoid pathway, which may contribute, at least in part, to the positive behavioral outcomes of NS treatment observed in this study (Figure 2, Figure 3 and Figure 4).

**Figure 8:**
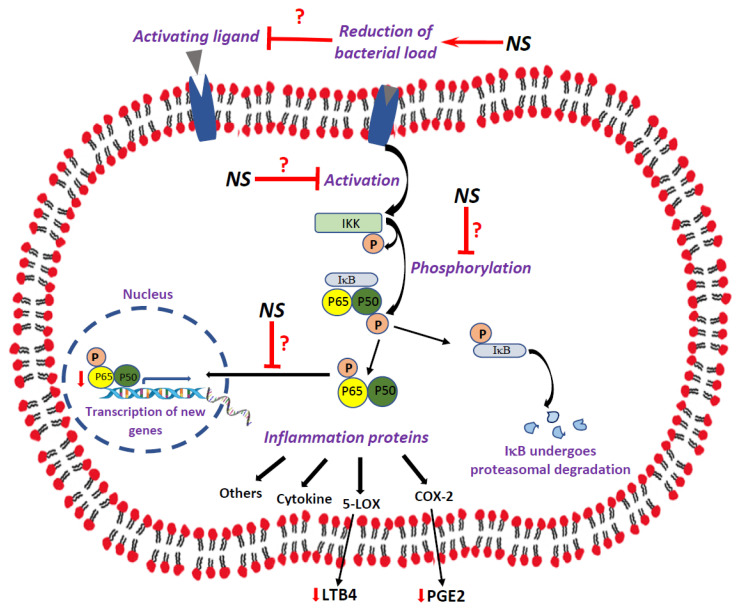
Possible mechanisms underlying the observed anti-inflammatory effects of NS. In this study, NS reduced nuclear P-p65 levels, which is indicative of the suppression of NF-κB activation. Additionally, NS decreased the levels of LTB4 and PGE2, which are produced by 5-LOX and COX-2, respectively. NS may impede the activation of NF-κB through the following postulated mechanisms: the (1) blocking of IKK activation; (2) inhibition of IKK activity; (3) prevention of P-p65-p50 penetration into the nucleus; (4) increment of IκB levels in the cytosol; (5) inhibition of bacterial-induced NF-κB activation through membrane receptors (due to the antimicrobial effects of NS); and (6) other impacts. 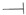 Indicates inhibition/blocking; ↓ indicates reduction; ? indicates a possible, not solidly proven effect. Abbreviations: COX, cyclooxygenase; IκB, inhibitor κ B; IKK, I-κB kinase; LOX, lipoxygenase.

Several studies have associated NF-κB to the pathophysiology and treatment of psychiatric illnesses [87,88,89,90,91,92]. Following the observation that NS suppresses the production of eicosanoids, and in a quest to find a possible mechanism that may explain these findings, we tested the impact of NS on the activation of the NF-κB pathway. As seen in Figure 7A, NS significantly decreased the nuclear P-p65 levels in the HT of male rats and non-significantly decreased these levels in female rats. On the other hand, NS treatment did not alter P-p65 levels in the HC (Figure 7B). These results are consistent with the findings of previous studies that demonstrated that NS reduces NF-κB activation and its related inflammation [13,30,31,118,128,129]. Moreover, they support our assumption that the inhibition of NF-κB activation may contribute to its suppressive effect on eicosanoid production. This is especially true because it was only in the HT of male rats where NS significantly diminished the levels of both PGE2 and LTB4 (Figure 6A,C), and also significantly mitigated nuclear P-p65 levels (Figure 7A). The precise mechanism underlying the inhibition of NF-κB activation as witnessed in the reduction in nuclear P-p65 is currently unknown. As illustrated in Figure 8, NS may inhibit NF-κB activation by altering the different steps of the activation cascade: (1) NS may impede the activation of IKK [130,131]; (2) NS may inhibit the catalytic activity of IKK [132,133]; (3) NS may hinder the entrance of the phosphorylated complex into the nucleus by blocking the nuclear localization sequence [134]; (4) NS may enhance the activity of IκB, similar to glucocorticoids [135]; (5) owing to the well-established association between the gut microbiome and neurological disorders [136], and considering the strong antimicrobial properties of NS (thymoquinone in particular) [7,102,137], it is possible that NS indirectly inhibits NF-κB by suppressing its activation through the bacterial-induced stimulation of membrane receptors; and (6) other.

Thus, according to the hypothesized mechanism and as illustrated in Figure 8, NS suppresses the expression of COX-2 and 5-LOX due to its inhibition of NF-κB. Although in the present study we did not assess the expression level (or activity) of COX-2 and 5-LOX, the observed reduction in PGE2 and LTB4 levels still supports our assumption. Consistently, Al Wafai found that NS and its active compound thymoquinone suppressed COX-2 expression in diabetic rats [138]. Similarly, it was reported that thymoquinone suppressed the formation of 5-LOX products (e.g., LTB4) in rats [125].

Our study has a limitation that is possibly one of the factors that led to some of the unexpected results that we obtained: we used naïve rats—not rats that were subjected to a stress protocol to induce a depression-like phenotype—to examine the antidepressive-like potential of NS. Thus, the effect of the NS treatment on sucrose consumption reflects a hedonic-like effect rather than an antidepressant-like effect. However, it is imperative to emphasize in this regard that the primary objective of the present study was to investigate the antimanic-like effect of NS, and not its antidepressant capacity, because the latter has been extensively studied in the past [9,11,61,62,95].

## 4. Materials and Methods

### 4.1. Animals

Male and female Sprague Dawley rats weighing 220–250 g at the beginning of the experiments were used in the study. Rats were housed three per cage under controlled conditions (ambient temperature 22 ± 1 °C, relative humidity 45–55%, photoperiod cycle 12 h light:12 h dark), with food and water ad libitum, unless otherwise indicated. The practices of the animal experiments were approved by the Committee for the Use and Care of Laboratory Animals at Ben-Gurion University of the Negev, Israel (Authorization # IL-48-10-2022D). At the inception of the experimental protocol, rats were randomly allocated to the various experimental groups. Modifications were made only to adjust for differences in the mean body weight of the groups.

### 4.2. Chronic Treatment with NS

Premium-grade NS oil—extracted through a cold-press manufacturing process—was procured from a commercially available vendor (Better Flax; Hadera, Israel). This selection was deliberate, aiming to employ an NS product with public availability and regular consumption. The nutritional value of the product (per 100 mL) as indicated by the manufacturer is as follows: Calories—745, protein—0 g, carbohydrates—0 g, total fat—92 g, saturated fat—20 g, monounsaturated fat—20 g, polyunsaturated fat—52 g, trans fat—<0.5 g, and cholesterol—<2.5 g. The content of thymoquinone in the NS oils varies greatly in different products [7,20,139,140], with the precise composition changing according to the NS species, seed type, and oil extraction technique. The content of thymoquinone in the oil used in the present study was determined using a gas chromatograph, similar to previous protocols [20,139]. The analysis revealed that the used oil contained 800 mg/100 g. This is consistent with the results of a previous study that examined the content of thymoquinone in multiple commercially available NS products [140]. Rats were fed for four weeks in accordance with two possible dietary conditions: regular powdered rodent chow (control group) or regular powdered chow enriched with NS oil [250 µg per kg of rat body weight] (treatment group). Weight-adjusted quantities of NS oil were freshly added to the powdered food of the treatment groups. On each day of the treatment protocol (unless otherwise indicated), animals were exposed to the study regimens after 10 h of food starvation, to make sure they consume all the NS-containing food. The NS-containing food was placed in specially designed containers situated to allow simultaneous and equalized access to all rats. Thereafter, all animals were given free access to regular food again. It is worth noting that a four-week treatment duration with rats parallels approximately two to six years in humans [141,142], resembling a chronic treatment/consumption protocol.

### 4.3. Behavioral Tests

All behavioral studies were conducted during the dark phase.

#### 4.3.1. Sucrose Consumption Test (SCT)

**Figure 9:**
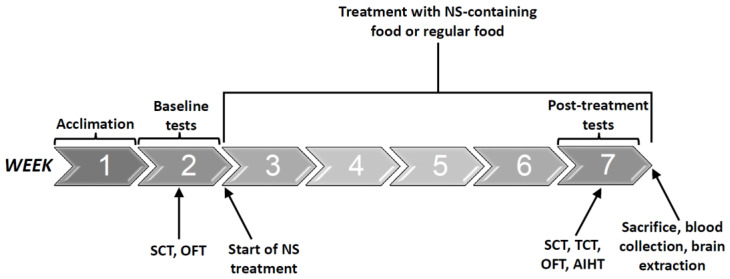
Timeline of the experimental protocol. Abbreviations: AIHT—amphetamine-induced hyperactivity test, NS—Nigella sativa, OFT—open field test, SCT—sucrose consumption test, TCT—three-chamber test. The time-order of the post-treatment behavioral tests was as follows: SCT, TCT, OFT, and lastly AIHT.

Generally, this test is used to assess anhedonia—a common behavioral manifestation in patients with depression. In the present study, given that we used naïve (“non-depressed”) rats, this test was orchestrated to assess hedonic-like behavior in the experimental animals. The test was conducted as described previously [143,144], with slight modifications. Sucrose consumption during a 24 h session was calculated as described previously [143,144]. Importantly, during the test sessions, rats had free access to the regular food chow and a bottle of water. The SCT was performed under identical conditions at two time points, as illustrated in Figure 9.

#### 4.3.2. Open Field Test (OFT)

This test is performed to assess the locomotor activity of rodents. The open field arena was made of a black box (60 cm [W] × 80 cm [L] × 60 cm [H]). Rats were placed in the arena for 30 min and sessions were videorecorded by a camera placed approximately one meter above the center of the arena. A 5% ethanol in water solution was used to wipe the arena prior to bringing in the next rat. Subsequently, sessions were evaluated utilizing a video-tracking system (Ethovision, XT 14; Noldus Information Technology, Wageningen, The Netherlands). Only the last 20 min of the sessions were analyzed; the initial 10 min were regarded as adaptation time. The measures that were evaluated for each session were the scalar mean velocity and total distance traveled [93,94,144].

#### 4.3.3. Three-Chamber Test (TCT)

This test examines rodents’ cognition in the mode of general sociability and interest in social novelty. Normally, rodents prefer to stay with each other rather than be alone (=sociability) and tend to explore a novel intruder when it is brought to the same arena (=social novelty). The three-chamber arena was made of a black box (40 cm [W] × 120 cm [L] × 60 cm [H]) divided into three equal chambers (squares). A test animal could freely access each of the three chambers as they are opened to each other through constantly opened “gates”. In the TCT, a test rat was initially placed in the central chamber, allowing it to explore the whole arena for five min. At this stage, each of the two external chambers contained an empty basket that the rat could sniff and explore. Then, a first new rat was put into one of the empty baskets, and the interaction of the test rat with the intruder was videotaped for 10 min. Thereafter, a second novel rat was brought to the other basket, and the interaction of the test rat with the newer rat was videotaped for 10 min. Thus, the measured parameters in the TCT were the duration of time spent by the test rat next to the first intruder (sociability), and the duration of time spent by the test rat next to the second intruder (social novelty).

#### 4.3.4. Amphetamine-Induced Hyperactivity Test (AIHT)

This is a broadly utilized model for measuring hyperactivity and mania-like behavior in rodents [93,94,145]. On the test day, rats were injected intraperitoneally with amphetamine 1 mg/kg (D-amphetamine sulfate, Bulk 281, Bio-Techne Ltd., Abingdon, UK), and then were returned to their home cage and allowed to stay in it for 30 min. Immediately thereafter, the rats were placed in an open field arena to measure their locomotor activity as described above (the tested measures were total distance traveled and mean velocity).

The chronological sequence of the experimental protocol is presented in Figure 2. Before the initiation of the behavioral studies, an acclimatization phase spanning one week was allotted to allow rats to adapt to standard housing conditions. Following this, baseline behavioral tests were conducted over the subsequent week. Thereafter, the treatment regimens were initiated and lasted for four weeks; during this period, aside from the daily 10-h food deprivation, rats were kept under normal housing conditions. After the completion of the treatment protocol, post-treatment behavioral tests were performed during the seventh week, at the end of which, the rats were euthanized for tissue collection.

### 4.4. Tissue Collection and Processing of the Samples

At the end of the experiment protocols (see Figure 9 for illustration), the rats were anesthetized with 4% isoflurane in 100% oxygen. Immediately after sacrifice, their brains were dissected for the extraction of the HT and hippocampus (HC), as described previously [93,143]. The HT regulates the production of several hormones and the activity of the immune system, affects a wide range of behavioral patterns, and maintains body homeostasis; the HC is a multi-functional part of the limbic system. It controls several visceral functions and influences other neurophysiological phenomena like memory, learning, and behavior [93,144]. The levels of IL-6, leukotriene (LT) B4, PGE2, and TNF-α in the brain samples were tested as described previously [45,93,94,145] using ELISA kits (*R&D Systems*, Minneapolis, Minnesota, USA). Subsequently, the brain samples were further processed to examine the levels of the phosphorylated form of the NF-κB protein p65 (P-p65) in the nuclear fraction of the samples using a specific ELISA kit (eBioscience, San Diego, CA, USA), as described previously [114]. Of note, increased levels of P-p65 in the nucleus is a marker of NF-κB activation [31,114,128,145].

### 4.5. Statistical Analyses

Firstly, normality tests were performed, revealing that all data were distributed normally. Accordingly, one-way ANOVA followed by post hoc Fisher’s LSD test were performed for between-group comparisons (male and female rats were analyzed separately). Values of *p* < 0.05 were considered statistically significant. Results are presented as mean ± SEM for the sample size, as indicated in each figure. Figure 2, Figure 3, Figure 4, Figure 5, Figure 6 and Figure 7 present the results of one out of two independent experiments demonstrating similar results. The total number of animals used in the entire study was 144 rats: *n* = 12 rats per group in the first experiment, and *n* = 24 rats per group in the second experiment.

## 5. Conclusions

The findings of the present study show that the chronic consumption of commercially available NS oil at “therapeutically relevant” amounts is associated with beneficial anti-inflammatory and behavioral outcomes, including an anti-manic-like effect, posing an additional facet to the therapeutic potential of this herb.

## Data Availability

Data are contained within the article.
